# Exploring drug combinations in genetic interaction network

**DOI:** 10.1186/1471-2105-13-S7-S7

**Published:** 2012-05-08

**Authors:** Yin-Ying Wang, Ke-Jia Xu, Jiangning Song, Xing-Ming Zhao

**Affiliations:** 1 Institute of Systems Biology, Shanghai University, Shanghai 200444, China; 2 Department of Mathematics, Shanghai University, Shanghai 200444, China; 3 Tianjin Institute of Industrial Biotechnology, Chinese Academy of Sciences, Tianjin 300308, China; 4 Department of Biochemistry and Molecular Biology, Monash University, Melbourne, VIC 3800, Australia

## Abstract

**Background:**

Drug combination that consists of distinctive agents is an attractive strategy to combat complex diseases and has been widely used clinically with improved therapeutic effects. However, the identification of efficacious drug combinations remains a non-trivial and challenging task due to the huge number of possible combinations among the candidate drugs. As an important factor, the molecular context in which drugs exert their functions can provide crucial insights into the mechanism underlying drug combinations.

**Results:**

In this work, we present a network biology approach to investigate drug combinations and their target proteins in the context of genetic interaction networks and the related human pathways, in order to better understand the underlying rules of effective drug combinations. Our results indicate that combinatorial drugs tend to have a smaller effect radius in the genetic interaction networks, which is an important parameter to describe the therapeutic effect of a drug combination from the network perspective. We also find that drug combinations are more likely to modulate functionally related pathways.

**Conclusions:**

This study confirms that the molecular networks where drug combinations exert their functions can indeed provide important insights into the underlying rules of effective drug combinations. We hope that our findings can help shortcut the expedition of the future discovery of novel drug combinations.

## Background

Drug combination refers to the combination of different agents that can achieve better efficacy but less side effects compared to any of its component drugs. Generally, drug combinations are composed of compounds that are single effective drugs. Recently, drug combination is becoming a promising and popular strategy in the clinic, especially in treating complex diseases such as cancer [1-3]. For example, DuoDote is the combination of Atropine and Pralidoxime, which is an approved combinatorial drug used to treat patients with antidote for organophosphorous [4]. Chan *et al*. [5] designed a combinatorial drug, namely Tri-Luma (containing fluocinolone acetonide, hydroquinone and tretinoin), for combating melasma (dark skin patches) of the face based on the efficacy and safety experiments. Agrawal *et al*. [6] found two effective combinatorial regimens to treat Huntington disease based on screening in *Drosophila*.

Despite these efforts that have been made to discover new drug combinations in the past few decades, a large portion of the effective combinatorial drugs clinically used were discovered through experiences that generally require labor-intensive and time-consuming "brute force" screening of all possible combinations between the approved individual drugs. When two drugs are administered together, one drug may promote or suppress the effect of the other. For instance, cyclosporine increases the effect of sirolimus, while bupropion decreases the effect of cyclosporine. As a result, the combination of two drugs may have a totally new effect that is different and unexpected from either individual drug [7,8]. Furthermore, the number of possible combinations will increase exponentially with the increasing availability of single drugs. For example, in the case of four drugs, there are six possible combinations. This number would be extremely large considering the fact that there are thousands of approved drugs. Due to the huge search space of possible combinations between known drugs, the identification of optimal and effective drug combinations is a challenging task.

Recently, a number of computational methods have been proposed to predict new drug combinations prior to combination synthesis and practical test in the lab. For example, Calzolari *et al*. [9] devised an efficient search algorithm originated from information theory to optimize drug combinations based on the sequential decoding algorithms. Geva-Zatorsky *et al*. [10] found that the protein dynamics in response to drug combination can be accurately described by a linear superposition of the dynamics under the corresponding individual drugs. In our recent work [11], we proposed a statistical model to predict new combinations based on a "drug cocktail" network constructed from effective drug combinations. We found that the effective combinations can be predicted based on the therapeutic effects of drugs and their interaction partners. Thanks to the completion of human genome sequencing projects, extensive systems biology efforts have been made to discover new combinations, especially from the network biology perspective in the past few years [11-14], from which the drug combinations can be identified based on the subnetworks or pathways perturbed by drugs [15,16]. Most recently, Zhao *et al*. [17] proposed a novel approach to predict drug combinations by integrating molecular and pharmlogical data, yielding promising results.

In general, one drug acquires the desired therapeutic effects by affecting the biological systems through the molecular pathways or biological processes pertinent to its target protein(s) [18]. For drug combinations, different component compounds may target different biochemical pathways to overcome the redundant processes dysregulated due to diseases. Therefore, the network circuits within which a drug functions should provide useful insights into the action mechanism underlying this drug, and thus help to understand the rules that underlie drug combinations. In this work, we explored drug combinations in the molecular space through the protein targets modulated by drugs. In particular, the proteins targeted by drugs were investigated in the context of genetic interaction map and pathway circuits. The genetic interactions were considered here because they are found important to drug actions [19] and causality of diseases [20]. Our results demonstrate that the proteins targeted by different compounds from one combination tend to have shorter distance within genetic interaction networks when compared with those of random combinations. Furthermore, drug combinations are found to be more likely to target the interacting pathways. We believe that the findings in this study can help to better understand the rules underlying drug combinations and are useful for predicting novel combinatorial drugs in the future.

## Results and discussion

To explore the effective drug combinations, a drug cocktail network was constructed following the same procedures as described in our previous work [11], where each node represented a drug while an edge was connected between the two drugs if they can be combined. In addition, we compared the effective combinations against the random combinations, which were generated by randomly shuffling the edges in the drug cocktail network while preserving the node degrees.

### Drug combinations tend to have shorter effect radius

**Figure 1:**
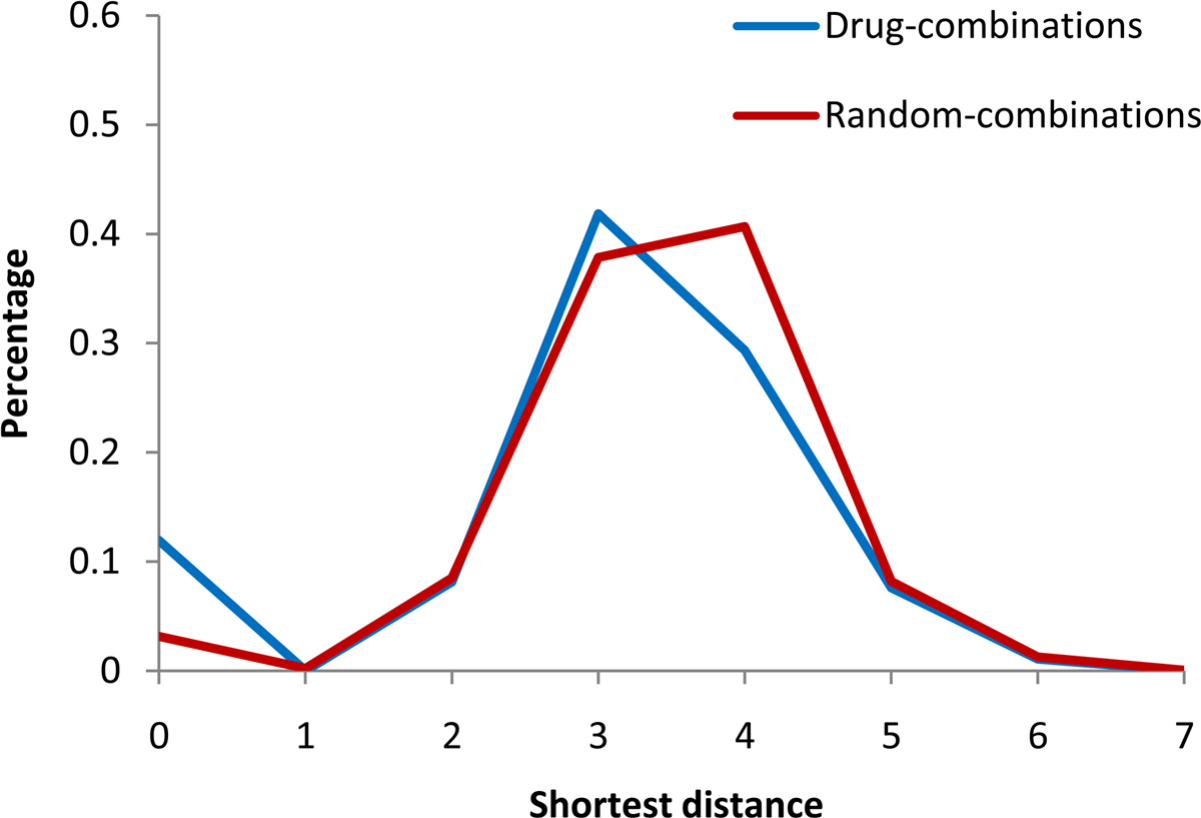
**The percentage of drug combinations with different effect radius**.

We firstly investigated the effect radius of both drug combinations and random combinations. Figure 1 shows the percentage of drug combinations with respect to their effect radius. Here, the genetic interactions between genes were considered because they are important for understanding drug combinations [19]. We can see that most drug combinations (62%) have short radius less than 3. Drug combinations have significantly shorter effect radius (*p*-value = 0.002 by the Wilcoxon test) compared with the random combinations, which implies that drug combinations may achieve their effects by modulating genetic interactions.

**Figure 2:**
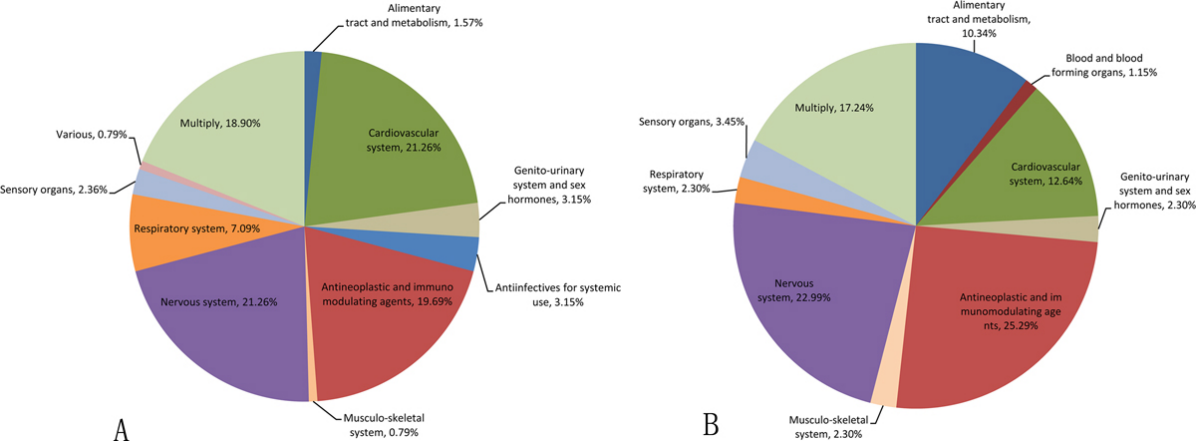
**The therapeutic categories of drugs involved in combinations with respect to their effect radius**. (A) Effect radius less than or equal to 3. (B) Effect radius larger than 3.

Furthermore, we investigated the therapeutic categories of the drugs invovled in combinations that have smaller effect radius (<=3) and those have larger effect radius (>3) (Figure 2). We can see that these two groups of drug combinations have some differences with respect to their therapeutic effects. For example, the drugs indicated for cardiovascular diseases and nervous diseases are the first two largest categories of drug combinations that have smaller effect radius, while the antineoplastic and immunomodulating agents are the largest group for drug combinations that have larger effect radius.

### Drug combinations tend to target the interacting and cross-talking pathways

**Figure 3:**
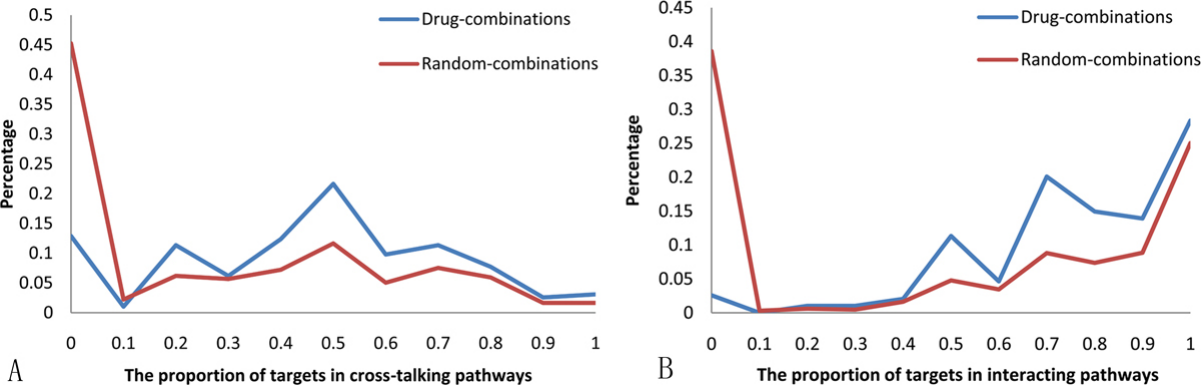
**The percentage of drug combinations with respect to the proportion of different types of pathway pairs targeted by the combinations**. (A) Cross-talking pathways. (B) Interacting pathways.

Using the target protein information, each drug was associated with the pathways in which its target proteins can be found. By grouping the relationships between the two pathways targeted by different agents of a combinatorial drug into four categories, i.e. interacting, cross-talking, identical, and parallel (see Methods), we found that drug combinations tend to significantly target the cross-talking (*p*-value 1.6e-45 by the Wilcoxon rank-sum test) and interacting pathways (*p*-value 4.3e-31 by the Wilcoxon rank-sum test) compared with the random combinations. Figure 3 shows the distribution of drug combinations according to the propotions of different pathway pairs targeted by the agents in the combination. In terms of the pathways targeted by drug combinations, we can see that most drugs are combined through the modulation of functionally related pathways, offering insightful clues to the identification of new drug combinations in the future. The dominant interacting pathways targeted by drug combinations also imply that genetic interactions may play important roles in drug combinations, which was confirmed recently [19].

**Figure 4:**
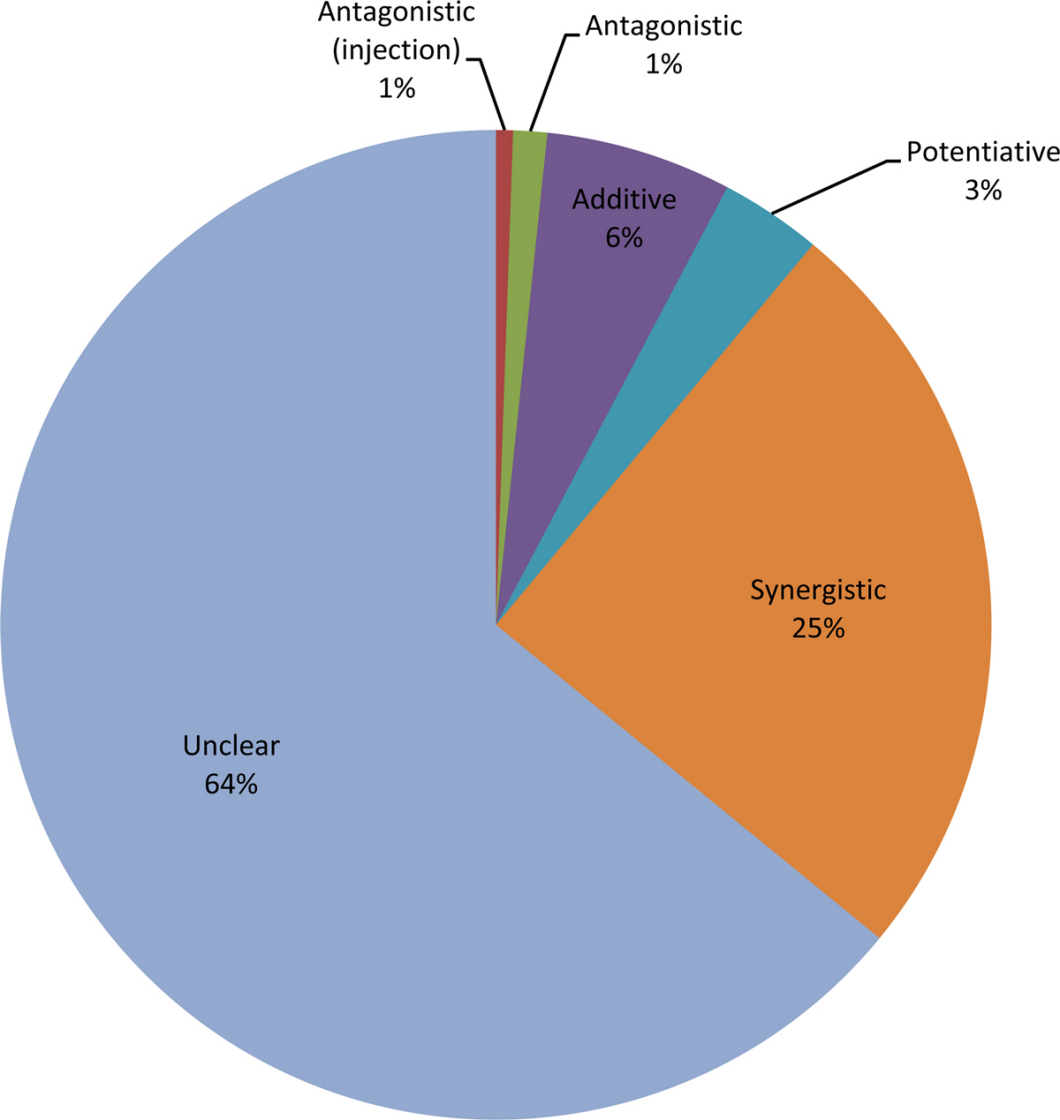
**Distribution of different types of drug combinations that target the interacting pathways**.

Specifically, we investigated the types of drug combinations that target interacting pathways. In the DCDB database, the drug combinations are roughly grouped into four categories: synergistic, additive, antagonistic and potentiative (details can be found in the DCDB database). Focusing on the drug combinations that target interacting pathways (i.e. drug combinations that have interacting pathways with the proportion larger than 0.4 in Figure 3A), we found that for the drug combinations that modulate interacting pathways, most are annotated as synergistic combinations as shown in Figure 4. In other words, drugs targeting distinctive but functionally related pathways may lead to synergestic therapeutic effects if they are used concurrently. We speculated that synergistic drug combinations may achieve their effects through modulating genetic interactions that bridge the related pathways. In general, the more pathways a drug affects, the more possible side effects it may introduce. The interacting pathways targeted by drug combinations may help to explain why synergistic drug combinations have higher selectivity or less side effects compared with their individual components [21].

**Figure 5:**
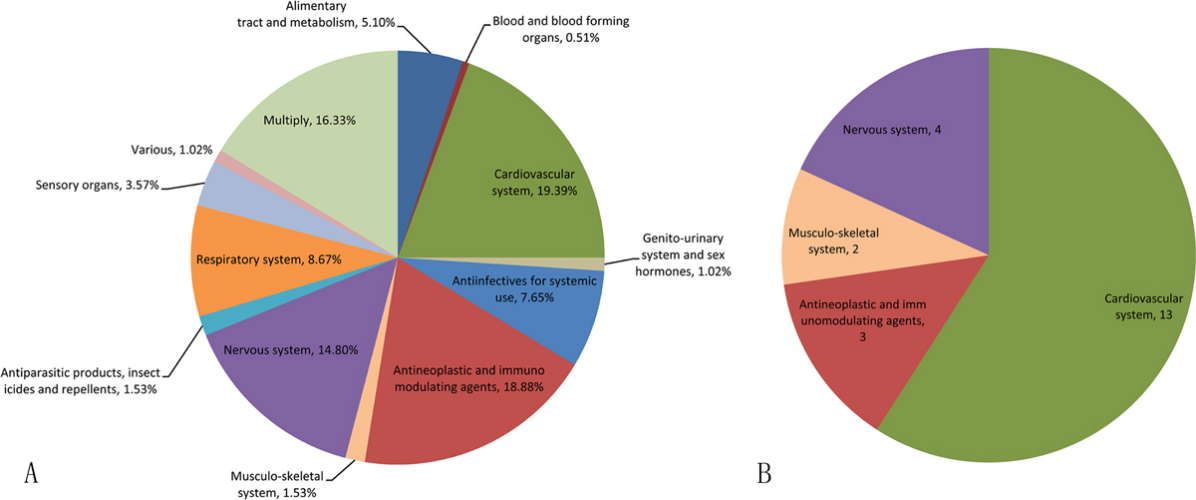
**The therapeutic categories of drugs involved in combinations with respect to the proportion of their targeted interacting pathways**. (A) Proportion of the interacting pathways larger than 0.4. (B) Proportion less than or equal to 0.4.

**Figure 6:**
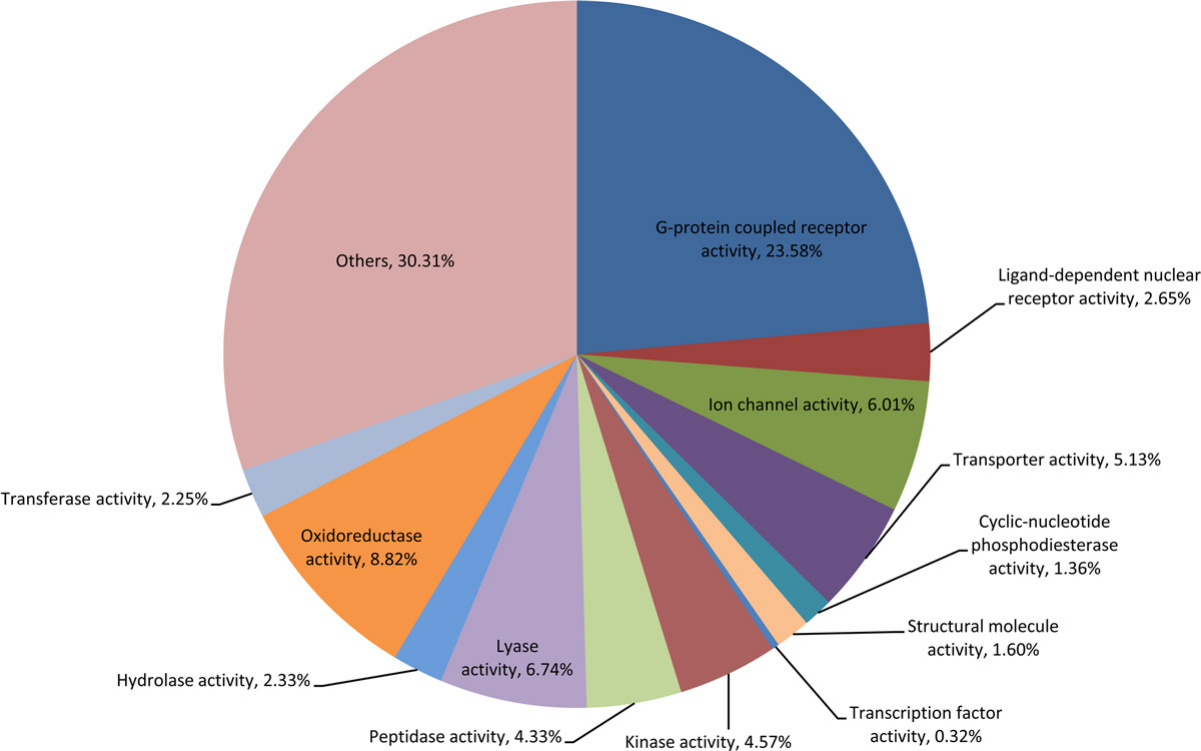
**The functional categories of proteins targeted by drug combinations that regulate the interacting pathways**.

Furthermore, we investigated the therapeutic effects of drug combinations that target the interacting pathways. Figure 5 shows the therapeutic categories of different drug combinations. There are 194 drug combinations that target interacting pathways, and most of the drugs involved are related to diverse disease types, including cardiovascular, neural and immune system related diseases. On the other hand, most of the drugs (59%) that do not target interacting pathways are indicated for cardiovascular diseases. In addition, we checked the proteins targeted by those drug combinations that tend to regulate interacting pathways (Figure 6). We found that these drug combinations modulate proteins with a broad range of biological functions, in which the G-protein coupled receptors (GPCRs) are predominantly involved. In summary, we found that the drugs involved in effective combinations that regulate interacting pathways are significantly different from those that do not tend to target interacting pathways, and further the former modulates proteins with a variety of biological functions.

## Conclusions

Drug combination is a promising strategy for combating complex disease, however, our understanding of mechanisms that underlie drug combinations is largely lacking at present. In this work, we explored drug combinations in the molecular space, especially in the context of genetic interaction networks and the pathways in which the target proteins function. By comparing the effective drug combinations and random combinations, we found that drug combinations tend to target proteins that are close in the genetic interaction networks. In addition, drugs that tend to modulate functionally related pathways or biological processes are likely to lead to synergistic interactions if they are administered concurrently. From our analysis, we conclude that the genetic interactions play important roles in drug combinations and can help to provide insights into mechanisms underlying drug combinations.

## Methods

### Data sources

The drug combinations were retrieved from a newly released Drug Combination Database (DCDB) [22], which is a major resource for collecting effective drug combinations from the literature. The target proteins and therapeutic (represented as Anatomical Therapeutic Chemical (ATC) classification system) information associated with the drugs were extracted from DrugBank [23]. In particular, the non-specific target proteins were discarded as described in [24]. Drug combinations that consist of drugs without the ATC annotation or target information were also discarded. Consequently, 444 effective drug combinations were obtained, including 182 approved, 54 clinical and 208 preclinical combinations. Based on these drug combinations, a drug cocktail network was constructed where each node represented a drug and an edge was added for the two drugs that were involved in the same combination. The random combinations were generated by randomly shuffling the edges in the drug cocktail network while preserving the degree for each node. This procedure was repeated for 1,000 times.

Human genetic interactions were obtained from [25] which were inferred by utilizing the radiation hybrid genotyping data. Here, only the interactions with *p*-value less than 1.0e-7 were considered. In addition, we obtained 234 pathways from the KEGG [26] database.

### The effect radius of a combinatorial drug

Given the drug components in a combination and their corresponding target proteins, the distance *dis*(*i,j*) between the two drugs *i *and *j *is defined as the shortest of the distances between the target proteins of drug *i *and *j*, where the distance between two proteins is calculated as the shortest path distance within the genetic interaction network. Wherever the distance between any two drugs is available, the effect radius of a combination drug can be defined as follows:

$$R=\frac{\underset{\left(i,j\right)\in D,\;i\neq j}{\sum }dis\left(i,j\right)}{\left|D\right|}$$

where *R *is the effect radius of one combinatorial drug, *dis*(*i,j*) denotes the distance between drugs *i *and *j*, *D *is the set of all possible combinations between the components of a drug combination, and |*D*| is the size of *D*, respectively.

### The interaction between pathways and drug combinations

For the two drugs in a combination, each can be associated with pathways to which its target proteins belong, and therefore two pathway sets are constructed for these two drugs. Given a pair of pathways that are respectively associated with these two drugs, we grouped this pathway pair into one of the following four categories: identical, cross-talking, interacting, and parallel and unrelated. The cross-talking pathways are those that have at least one common gene, the interacting pathways have genetic interaction(s) between the two pathways, while the parallel and unrelated pathways consist of two totally different pathways without any functional relationships. For a given drug combination, we investigated the proportion of the four types of pathway pairs modulated by the combination.

## Competing interests

The authors declare that they have no competing interests.

## Authors' contributions

YYW and KJX implemented the computational methods, carried out data analysis, and drafted the manuscript under the direction of XMZ and JS. XMZ guided and coordinated the project. XMZ and JS improved the presentation of the manuscript by copy-editing and fixing language issues. All authors read, revised and approved the final manuscript.
